# Improvement of Bipolar Switching Properties of Gd:SiO_x_ RRAM Devices on Indium Tin Oxide Electrode by Low-Temperature Supercritical CO_2_ Treatment

**DOI:** 10.1186/s11671-016-1272-5

**Published:** 2016-02-01

**Authors:** Kai-Huang Chen, Kuan-Chang Chang, Ting-Chang Chang, Tsung-Ming Tsai, Shu-Ping Liang, Tai-Fa Young, Yong-En Syu, Simon M. Sze

**Affiliations:** Department of Electrical Engineering and Computer Science, Tung Fang Design Institute, Kaohsiung, Taiwan, Republic of China; Department of Materials and Optoelectronic Science, National Sun Yat-Sen University, Kaohsiung, Taiwan, Republic of China; Department of Physics, National Sun Yat-Sen University, Kaohsiung, Taiwan, Republic of China; Advanced Optoelectronics Technology Center, National Cheng Kung University, Tainan, Taiwan, Republic of China; Department of Mechanical and Electro-Mechanical Engineering, National Sun Yat-Sen University, Kaohsiung, Taiwan, Republic of China; Department of Electronics Engineering and Institute of Electronics, National Chiao Tung University, Hsinchu, Taiwan, Republic of China

**Keywords:** Nonvolatile memory, Gadolinium, Supercritical CO_2_, Resistive switching, Silicon oxide

## Abstract

Bipolar switching resistance behaviors of the Gd:SiO_2_ resistive random access memory (RRAM) devices on indium tin oxide electrode by the low-temperature supercritical CO_2_-treated technology were investigated. For physical and electrical measurement results obtained, the improvement on oxygen qualities, properties of indium tin oxide electrode, and operation current of the Gd:SiO_2_ RRAM devices were also observed. In addition, the initial metallic filament-forming model analyses and conduction transferred mechanism in switching resistance properties of the RRAM devices were verified and explained. Finally, the electrical reliability and retention properties of the Gd:SiO_2_ RRAM devices for low-resistance state (LRS)/high-resistance state (HRS) in different switching cycles were also measured for applications in nonvolatile random memory devices.

## Background

Many nonvolatile memory devices for ferroelectric random access memory (FeRAM), magnetic random access memory (MRAM), and phrase change memory (PCM) are widely discussed for applications in the smart memory cards, electronic devises, and portable electrical devices [1–8]. Among these memory devices, various metals doped into silicon-based oxide thin films are widely and considerably discussed for the resistive random access memory (RRAM) devices because of its great compatibility in integrated circuit (IC) processes, high operation speed, long retention time, and low operation voltage [9–13]. Recently, the transparent ITO electrode of the various memory devices are widely discussed and investigated because of its compatibility and integrated in system on panel concept applications [14–17]. The high thermal budget and fabrication cost of rapid temperature annealing (RTA) and conventional furnace annealing (CFA) post-treatment methods were widely used for applications in dielectric thin films reformed and passivated the defects [15–18]. However, the excellent liquid-like properties of the supercritical CO_2_ fluid (SCF) process have attracted considerable research in efficiently transporting H_2_O molecules diffusion into the microstructures of thin films at a low-temperature treatment [19–21].

To discuss the SCF-treated ITO electrode on bipolar switching properties of RRAM devices, the ITO/Gd:SiO_2_/TiN structure was treated by low-temperature SCF treatment. In addition, the electrical transferred conduction mechanism of the initial metallic filament-forming model was explained to bipolar switching properties of RRAM devices on ITO electrode in this study.

## Methods

The metal-insulator-metal (MIM) structure of Gd:SiO_2_ thin film RRAM devices was fabricated and prepared by SiO_2_ and gadolinium co-sputtering technology on the TiN/Ti/SiO_2_/Si substrate. The sputtering power was fixed with an rf power of 200 W and a DC power of 10 W. The 200-nm-thick ITO electrode was deposited on Gd:SiO_2_ film to form ITO/Gd:SiO_2_/TiN structure. In addition, the ITO/Gd:SiO_2_/TiN structure sample was placed in the supercritical fluid system, which was mixed with 5 vol.% pure H_2_O and 5 vol.% propyl alcohol, injected at 3000 psi and 150 °C for 2 h. The bipolar switching operation current versus applied voltage (*I*–*V*) characteristics of Gd:SiO_2_ RRAM devices are measured by Agilent B1500 semiconductor parameter analyzer. The X-ray photoelectron spectroscopy (XPS) is used to analyze the chemical composition and bonding of thin films, respectively.

## Results and Discussion

**Fig. 1:**
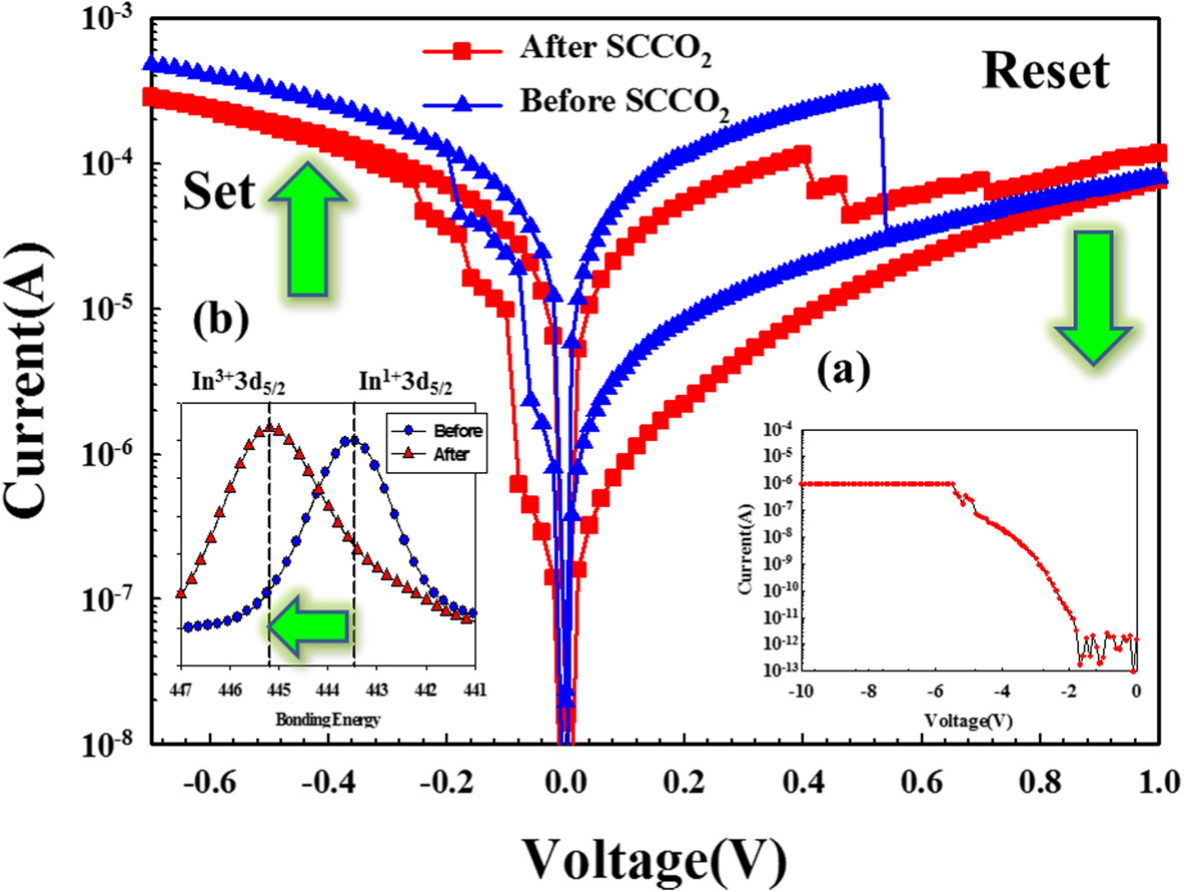
The typical *I*–*V* switching characteristics of the Gd:SiO_2_ thin film RRAM devices for (*a*) the initial forming process and (*b*) In^3+^3d_5/2_ of ITO electrode in XPS spectra

To investigate the SCF-treated ITO electrode effect, the bipolar resistance switching behavior of the Gd:SiO_2_ RRAM devices was discussed and observed in Fig. 1. After the initial forming process of −10 V in Fig. 1*b*, the Gd:SiO_2_ RRAM devices exhibited a low-resistance state (LRS). Then, a high-resistance state (HRS) was forming by high negative bias. To define the set process state, the RRAM devices exhibited the LRS for applying a large negative bias than the set voltage. For reset process state, a gradual current decrease was presented in LRS to HRS for the bias to positive over the reset voltage. For inverted set/reset state properties of the Gd:SiO_2_ RRAM devices, we suggested the transferred electron early captured by the lots of oxygen vacancy in top ITO electrode and formed the oppositely metallic filament [22]. The operation current of the Gd:SiO_2_ RRAM devices for using SCF-treated ITO electrode was lower than that for the nontreated electrode of others. In order to further discuss the initial metallic filament path diagram model, the electrical transferred mechanisms of RRAM devices for the SCF-treated ITO electrode were discussed and investigated.

**Fig. 2:**
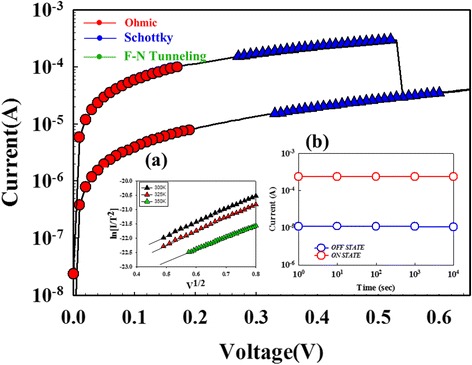
The *I*–*V* switching curves of the Gd:SiO_2_ RRAM devices using SCF-treated ITO electrode for LRS/HRS state in set state. (*a*) ln(I/T^2^)-V^1/2^ curve fitting and (*b*) the reliability properties for different switching cycle

**Fig. 3:**
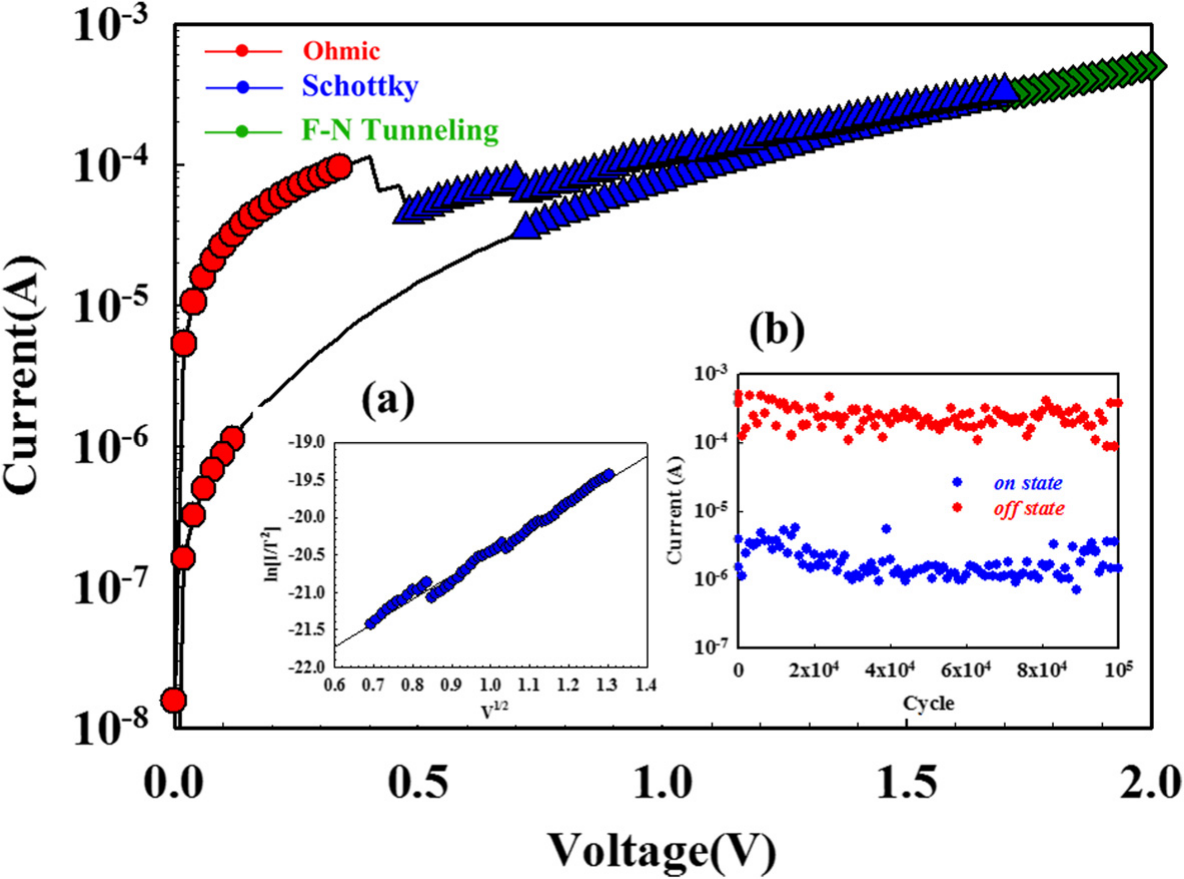
The *I*–*V* switching curves of the Gd:SiO_2_ RRAM devices using SCF-treated ITO electrode for LRS/HRS state in reset state. (*a*) ln(I/T^2^)-V^1/2^ curve fitting and (*b*) the retention characteristics for different switching cycling

According to the relationship of the Schottky emission equation, $$ J=A*{T}^2 \exp \left[-q\left({\phi}_{\mathrm{B}}-\sqrt{\raisebox{1ex}{$q{E}_{\mathrm{i}}$}\!\left/ \!\raisebox{-1ex}{$4\uppi {\varepsilon}_{\mathsf{i}}$}\right.}\right)/KT\right] $$, where *T* is the absolute temperature, *Φ*_B_ is the Schottky barrier height, *ε*_i_ is the insulator permittivity, *K* is Boltzmann’s constant, and A* is Richardson constant. The *I–V* switching curve of the Gd:SiO_2_ RRAM devices was transferred to ln(*I*/*T*^2^) − *V*^1/2^ and ln(*I*) − ln(*V*) curve to fit the Schottky emission and the ohmic conduction mechanism. In Fig. 2, the Gd:SiO_2_ RRAM devices for LRS/HRS in the set state exhibited the ohmic conduction mechanism for low applied voltage. In Fig. 2*a* for 0.3~0.5 V, the LRS/HRS of Gd:SiO_2_ RRAM devices all exhibited the Schottky emission conduction by ln(*I*/*T*^2^) − *V*^1/2^ curve fitting for the temperature of 300–350 K [23, 24]. If the *J*–*E* curves obey the Schottky emission model, the fitting curves should be straight in this figure. In Fig. 3*a*, the LRS/HRS of Gd:SiO_2_ RRAM devices in the reset state also exhibited the ohmic conduction mechanism by ln(*I*) − ln(*V*) curve and the Schottky emission conduction mechanism by ln(*I*/*T*^2^) − V^1/2^ curve fitting.

To analyze the oxygen element of the chemical composition characteristics in ITO electrode, the mole fraction of stannum (Sn), indium (In), and oxygen (O), in the ITO thin film was 5.08, 47.76, and 47.15 %, respectively, calculated from the peak areas of XPS spectra. For the SCF-treated ITO electrode, we found that the mole fraction of Sn, In, and O elements was 4.7, 18.32, and 76.98 %, respectively. The mole fraction of the oxygen element increased from 47.15 to 76.98 %. The increase of oxygen ion qualities and decrease of the electric conductivity of SCF-treated ITO electrode were also proved and verified in the XPS spectra. In Fig. 1*b*, the In^1+^3d_5/2_ peaks of ITO electrode that shifted two valences to In^3+^3d_5/2_ effect was caused and improved by oxidation ability and binding energy of SCF treatment. The oxidation ability and repaired damaged effect of ITO electrode of Gd:SiO_2_ RRAM devices improved by SCF treatment process were found [15–17].

**Fig. 4:**
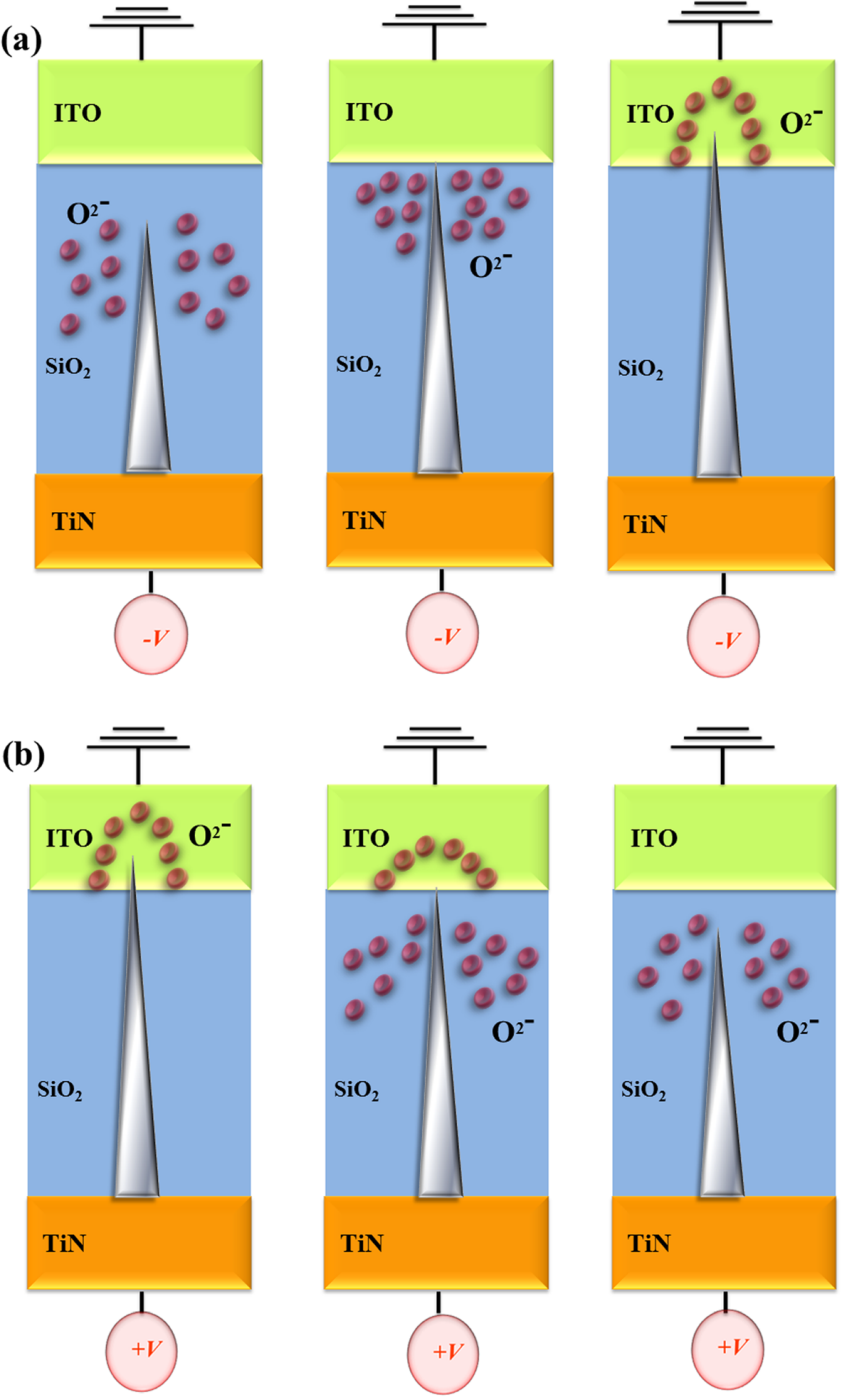
The electrical transferred mechanisms and metallic filament path diagram of the Gd:SiO_2_ RRAM devices using SCF-treated ITO electrode for **a** set state under the negative voltage and **b** reset state under the positive voltage

As discussed above, the electrical transferred mechanisms of *I*–*V* curves results, the metal filament path diagram model of the Gd:SiO_2_ RRAM devices was described. To the initial metallic filament path-forming process for the negative applied voltage, the uniform oxygen ions existed in Gd:SiO_2_ thin film of the RRAM devices for the set state are shown in Fig. 4a. To continuously apply negative voltage, lots of oxygen ions were accompanied into the ITO electrode. The metallic filament path increased and exhibited Schottky emission conduction mechanism. In Fig. 4b, the oxygen ions in ITO electrode return back to Gd:SiO_2_ thin film for the initial reset state exhibited the ohmic conduction mechanism for the low voltage applied. Then, the metallic filament path was decreased by oxygen ion oxidation and exhibited Schottky emission conduction mechanism for continuously applying positive voltage.

For the electrical reliability properties, the on/off ratio in *I*–*V* curves of the Gd:SiO_2_ RRAM devices was measured and obtained for the different switching cycle. In Fig. 2*b*, no significant changes in the current values for 10^4^ s were observed. In addition, the switching cycling measured another type of the retention characteristics shown in Fig. 3*b*. The slight fluctuation of the resistance in the LRS/HRS and the stable switching property of 10^5^ cycles exhibited the reliability properties of the nonvolatile Gd:SiO_2_ RRAM devices applications.

## Conclusions

In conclusion, the bipolar resistance switching characteristics and low power consumption of Gd:SiO_2_ RRAM devices for ITO top electrode were achieved by using a low-temperature supercritical CO_2_ treatment. The switching resistance mechanisms in the SCF-treated ITO electrode of RRAM devices for HRS/LRS were proved and investigated by electrical transferred mechanisms and a metallic filament path diagram model. Finally, no significant changes of the operation current of the electrical reliability properties in Gd:SiO_2_ RRAM devices for on/off state were maintained to 10^4^ s. For the retention characteristics, the slight fluctuation of resistance in the LRS/HRS states and the stable switching property of 10^5^ cycles were also found.
